# A multi-pronged scoping review approach to understanding the evolving implementation of the Smallpox and Polio eradication programs: what can other Global Health initiatives learn?

**DOI:** 10.1186/s12889-020-09439-1

**Published:** 2020-12-18

**Authors:** Meike Schleiff, Adetoun Olateju, Ellie Decker, Abigail H. Neel, Rasheedat Oke, Michael A. Peters, Aditi Rao, Olakunle Alonge

**Affiliations:** International Health Department, Johns Hopkins Bloomberg School of Public Health, 615 N. Wolfe St, Baltimore, MD 21205 USA

**Keywords:** Polio, Smallpox, Eradication, Implementation research, Scoping review, Systematic review, Protocol

## Abstract

**Background:**

Previous initiatives have aimed to document the history and legacy of the Smallpox Eradication Program (SEP) and the Global Polio Eradication Initiative (GPEI). In this multi-pronged scoping review, we explored the evolution and learning from SEP and GPEI implementation over time at global and country levels to inform other global health programs.

**Methods:**

Three related reviews of literature were conducted; we searched for documents on 1) the SEP and 2) GPEI via online database searches and also conducted global and national-level grey literature searches for documents related to the GPEI in seven purposively selected countries under the Synthesis and Translation of Research and Innovations from Polio Eradication (STRIPE) project. We included documents relevant to GPEI implementation. We conducted full text data analysis and captured data on Expert Recommendations for Implementing Change (ERIC) implementation strategies and principles, tools, outcomes, target audiences, and relevance to global health knowledge areas.

**Results:**

200 articles were included in the SEP scoping review, 1885 articles in the GPEI scoping review, and 963 documents in the grey literature review. M&E and engagement strategies were consistently translated from the SEP to GPEI; these evolved into newer approaches under the GPEI. Management strategies including setting up robust record systems also carried forward from SEP to GPEI; however, lessons around the need for operational flexibility in applying these strategies at national and sub-national levels did not. Similarly, strategies and lessons around conducting health systems readiness assessments prior to implementation were not carried forward from SEP to GPEI. Differences in the planning and communication strategies between the two programs included fidelity to implementation blueprints appeared to be higher under SEP, and independent monitoring boards and communication and media strategies were more prominent under GPEI.

**Conclusions:**

Linear learning did not always occur between SEP and GPEI; several lessons were lost and had to be re-learned. Implementation and adaptation of strategies in global health programs should be well codified, including information on the contextual, time and stakeholders’ issues that elicit adaptations. Such description can improve the systematic translation of knowledge, and gains in efficiency and effectiveness of future global health programs.

## Background

In recent decades, numerous factors—including social, political, economic, and environmental factors—have influenced global perspectives on the practicality, desirability, and approaches of eradication initiatives. Two global eradication efforts, the Smallpox Eradication Program (SEP) and the Global Polio Eradication Initiative (GPEI), offer important lessons on implementation strategies for addressing challenging factors to global eradication and other global health intiatives. This paper will explore how SEP and GPEI have utilized implementation strategies to further their eradication goals, and how some of these implementation strategies have evolved over time.

The successes and learnings from the Smallpox Eradication Program (SEP), the Expanded Program on Immunization (EPI) and initial polio eradication efforts in the Philippines, Brazil, and Cuba garnered optimism and focused efforts from Rotary International and the Pan-American Health Organization (PAHO) to prioritize polio eradication efforts [1, 2]. In 1981, PAHO concluded the elimination of polio was feasible, and with the support of the United Nations International Children’s Emergency Fund (UNICEF), launched an initiative to eradicate polio from the Americas in 1985 [2, 3]. Realizing the complexity and spread of the disease, the World Health Assembly subsequently launched the Global Polio Eradication Initiative (GPEI) in 1988 [4]. PAHO effectively eliminated polio in the Americas by 1994, and was the first WHO region to do so, [5] while other WHO regions then grew motivated and followed suit. The global effort has since lowered the incidence of polio by over 99% [6].

The SEP, launched in 1959 by the World Health Organization (WHO), faced resource limitations and lack of commitment from countries early on and had not achieved much progress towards elimination in Africa, Asia, or South America by 1966 [7–10]. In 1967, a re-vamped Intensified Eradication Program was initiated, which included an expansion of vaccine manufacturing capacity in endemic countries and a heightened focus on developing disease surveillance systems in countries where eradication activities were being implemented, in addition to the ongoing mass vaccination campaign strategy [11, 12]. At the same time, the development of the bifurcated needle improved the effectiveness of vaccine delivery and key strategies including efforts to achieve universal vaccination, surveillance-containment approaches where known or suspected contacts were rapidly identified and isolated, case identification, quarantine, contact tracing, primary vaccination, and ring vaccination strategies were deployed [9, 13–16]. By the mid-1970s, final cases were detected and efforts to certify the world free of smallpox were underway. On May 8, 1980 the 33rd World Health Assembly officially declared the world free from smallpox, a momentous achievement for global public health [8, 14].

The SEP demonstrated the promise of health programs targeting vaccine-preventable diseases to dramatically reduce the burden of communicable diseases globally, but the SEP experience also brought to light serious health system inadequacies which needed to be addressed for immunizations to be delivered at scale [9]. Recognizing this, in the final years of the SEP, the World Health Organization launched the EPI in 1974 [9]. The EPI prioritized immunization against other diseases such as diphtheria, measles, and polio, gradually shifting focus from the campaign-based strategy of the SEP to delivery through routine services. Inadequate equipment, lack of governmental and public awareness, and insufficient monitoring systems continued to challenge the EPI program; still, the EPI progressed, including greater emphasis on providing immunizations for diseases, through routine health services and increasing coverage of a package of vaccines to larger proportions of children [9, 17]. More resources were needed for this program, and WHO and UNICEF both provided substantial investments, under the leadership of WHO Director General, Halfdan Mahler, in 1977, and UNICEF’s executive director, James Grant, shortly thereafter [16, 18].

Of the vaccine-preventable diseases, polio attracted much attention given the debilitating nature of the illness among children and had champions spearheading efforts to eliminate poliovirus from the United States, which was achieved in 1979 [19]. Given the initial successes in reducing the burden of polio in the United States and Europe between 1955 (introduction of IPV in the US) and 1960s [20] through routine immunization was not successfully replicated in the lower-resource countries, Sabin recommended using mass immunizations to supplement routine immunizations [21]. The approach, first adopted by Cuba on national scale [16] and in pilots in Brazil in the 1960s and later nationally in the 1980s [22] along with other countries in the Americas culminated in PAHO setting forth a goal to eliminate polio across the Americas by 1990 [23]. PAHO countries strengthened their laboratory networks and, perfected the concept of mass immunization campaigns called “national immunization days” (NIDs) starting in Brazil in 1980 and subsequently adopted as PAHO’s model for eradication in the Americas [22]. Intercountry cooperation also played a key role in PAHO’s success by synchronizing efforts and bolstering shared motivation towards the common goal of eliminating poliovirus from the region [7].

Given PAHO’s success as well as advocacy efforts led largely by Rotary International, the World Health Assembly passed a resolution in 1988 to globally eradicate polio by the year 2000 and launched the Global Polio Eradication Initiative (GPEI) [4, 24]. Core partners of the GPEI included the World Health Organization (WHO), the US Centers for Disease Control and Prevention (CDC), Rotary International, and the United Nations Children’s Fund (UNICEF) at inception, and were later joined by the Bill and Melinda Gates Foundation (BMGF) in 2000, and Gavi, The Vaccine Alliance, in 2019. The partners have worked with governments and other implementers in more than 200 countries and territories to vaccinate over 2.5 billion children globally since 1988.(8) Although the eradication goal set for 2000 has not been achieved, the GPEI has made remarkable progress in reducing the global incidence of polio by over 99% as of early 2020 [6, 24].

The GPEI was established with a four-pronged eradication approach which was developed in the PAHO region as part of its regional effort to eradicate polio, and which drew upon tactics utilized in both the SEP and EPI. These prongs included: surveillance, routine immunization, supplementary immunization activities (SIAs), and targeted mop-up campaigns for reaching high-risk populations. Importantly, when the GPEI drafted it’s an initial strategic plan it was felt by partners the “specialized effort” required for eradication could be used as “leading edges” for strengthening routine immunization and primary health care more broadly [25]. This reflected the PAHO experience in which routine immunization served as the backbone of the eradication effort, as well as the relative success of the EPI in the preceding years to expand immunization coverage (at the time, the EPI was targeting 80% DTP3 coverage globally) [26]. While this four-pronged strategy has remained over the course of the GPEI, the balance of these strategies and the implementation mechanisms required to achieve each have necessarily shifted, expanded, and increased in precision over time. These adaptations have been in response to emerging implementation challenges – some of which are specific to the complexities of eradicating wild poliovirus – but also reflect key operational lessons learned over the course of the SEP, EPI, and early polio programs. These include the need for setting measurable objectives, evaluating progress, establishing quality control mechanisms for vaccines, ensuring highly qualified staff, and maintaining an active research agenda to inform implementation and provide evidence to solve outstanding questions [27].

As part of the Synthesis and Translation of Research and Innovations from Polio Eradication (STRIPE) consortium convened by The Johns Hopkins School of Public Health with partners from seven selected countries (Afghanistan, Bangladesh, Democratic Republic of the Congo, Ethiopia, India, Indonesia, and Nigeria), we implemented a multi-pronged scoping review to explore how global eradication strategies evolved from the SEP to the GPEI. To ensure representativeness, the countries were selected to represent the different epidemiological classifications for polio (endemic, outbreak, at-risk and polio-free), different geographical regions for the GPEI program, country income classifications, conflict-affected compared to stable countries, and countries that are regional leaders and representing large population. Our aim was to understand why some strategies were readily translated and others were not, and the role that people, context, and time played in such translation, or lack thereof. We hope to draw lessons for future global eradication programs and other efforts to respond to disease emergencies and widespread epidemics, and strengthen health systems globally.

While there have been other initiatives to document the histories of the smallpox and polio eradication programs, [13, 23, 28–38] our focus is to apply an implementation science lens to explore in detail how both the SEP and the GPEI utilized specific strategies to implement their programs. By considering the actors, processes, and contexts for conducting program activities, and how interactions between these forces contribute to program successes or failures, this study contributes a new perspective on these eradication initiatives. This holistic approach can help to synthesize and understand the contributors and barriers to program success and facilitate the application of relevant lessons to other eradication initiatives, broader public health programs, and across multiple disciplines to improve program design and implementation, ultimately contributing to better population health [39–41]. Accordingly, in this review study we have drawn upon concepts, theories, and frameworks from the interdisciplinary field of implementation science to understand the strategies, outcomes, tools, and principles/recommendations across the span of smallpox and polio eradication activities. Specifically, we reviewed implementation strategies documented from the SEP and the strategies and experiences under the GPEI using a standardized set of implementation science strategies from the Expert Recommendations for Implementing Change (ERIC) project [42], implementation outcomes [43], contextual factors from the Consolidated Framework for Implementation Research (CFIR) [44], and a recent review that defined different global health knowledge areas [45].

## Methods

### Conceptual framework

**Fig. 1:**
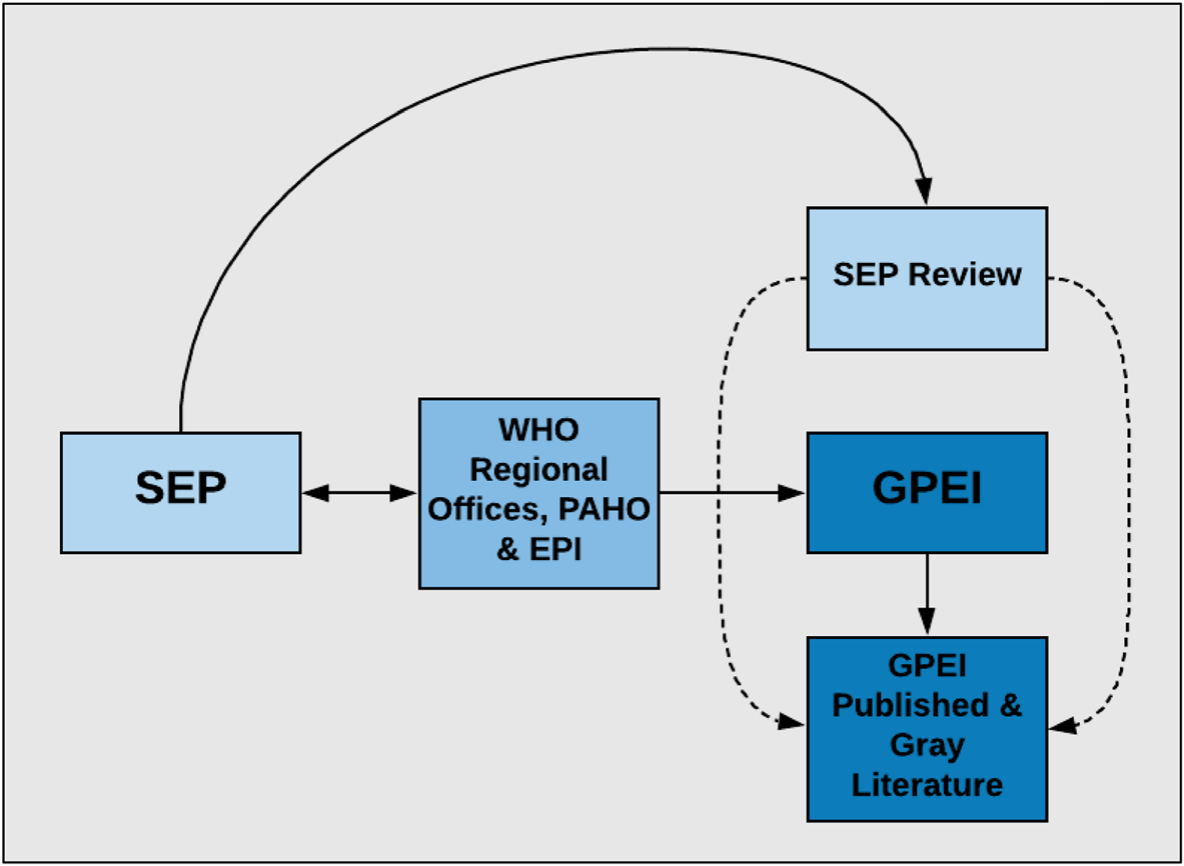
Conceptual Framework for the multi-pronged scoping review

The conceptual framework (Fig. 1) depicts how we posit the three prongs (SEP published literature review, GPEI published literature review, and GPEI grey literature review) of this scoping review are interconnected and the underlying rationale for including all three. Beginning from the top with the SEP review, the experiences from this first successful global eradication initiative have informed implementation strategies and explicit knowledge in both the published and grey literature of the GPEI. (shown by the solid arrows). The influence of the SEP on GPEI (shown by unidirectional dotted lines) can be captured from a scoping review of published literature —including perspectives from academia, global partnerships, and also the donor community— and grey literature—including perspectives from governments, policy makers, and implementing partners. The grey literature includes documents produced by multiple stakeholders of sufficient quality to be preserved, but not controlled by commercial publishers [46]. We posit that there has been bi-directional learning between the material that is reflected in the grey literature and published literature from the GPEI and this ongoing relationship between the two is depicted by bidirectional dotted lines.

This conceptual framework will be referenced in the following sections that further explore the search strategy and process of reviewing the material that were identified.

### Search strategy

#### Published literature

The SEP and GPEI published literature searches were similar and designed to capture the experience of implementing the SEP and the GPEI over time, including implementation strategies, tools, and lessons learned. For the SEP review, we searched PubMed, Scopus, and EMBASE; for the GPEI review, we restricted our search to PubMed given the relevance and quantity of material retrieved. Additional details regarding the search strategy are available in the Supplementary Materials file.

#### Grey literature

We included global-level as well as country-level grey literature. The global-level grey literature review aimed to trace major shifts, milestones, and key events related to the GPEI at the global level and provide an overall framing and tracing of the evolution of the GPEI for the rest of the GPEI literature to be contextualized within. Further details about the grey literature search process are available in the Supplementary Materials file.

### Inclusion/exclusion criteria and title/abstract screening

We developed the inclusion criteria for each prong of the review as summarized in Table 1. Exclusion criteria were defined as all materials that did not meet this set of criteria. Where lack of clarity emerged in the inclusion criteria related to the SEP and GPEI implementation, we reviewed as a team and made determinations based on relevance to the study objectives. We generally excluded articles that reported findings upstream of smallpox and polio eradication activities (i.e. disease physiology, vaccine development studies) or where there was no explicit link between the study and implications for program delivery (i.e. brief mentions of the importance of SEP or GPEI, but no details on how it was done or what should be learned from these initiatives). We also excluded articles discussing post-eradication considerations such as bioterrorism threats, and articles that reported studies on packages of vaccines in which polio was not a specific focus.

**Table 1:** Inclusion Criteria Across All Prongs of the Review

	SEP	GPEI	Grey Literature
**Relevant for understanding implementation of SEP/GPEI**	Describes implementation of the SEP (excludes lab-only studies, vaccine development studies)	Describes implementation of the GPEI (excludes lab-only studies, vaccine development studies)	Describes implementation of the GPEI (excludes lab-only studies, vaccine development studies)
**Time period**	January 1, 1950 to May 7, 2018	January 1, 1988-April 18, 2018	January 1, 1988 through end date for each country (between August 2018 and February 2019)
**Language**	English	English	English and languages in seven countries that teams were able to translate to English data
**Geographical area**	Global, all LMIC* AND all PAHO countries	Global, or all LMIC*	Global, or limited to seven countries, but included multi-country studies that included one of the seven

*Low- and Middle-Income (LMIC) countries as per 2017 World Bank classifications

Time periods of publication were selected to cover the entirety of the SEP and GPEI programs, respectively. We included articles in English for the published literature on SEP and GPEI; we also included articles in local languages for the grey literature across the seven countries. We had originally aimed to include Spanish and Portuguese articles in the SEP review due to the important leadership role of countries in the PAHO region but determined the cost and time required to complete this step with high quality translation and extraction of material was prohibitive.

Title and abstract screening was completed in Covidence (Melbourne, Australia) by two independent reviewers for each article. Local researchers within the STRIPE consortium completed reviews of the grey literature in their country contexts. Full details about the document screening process can be found in the Supplementary File.

**Fig. 2:**
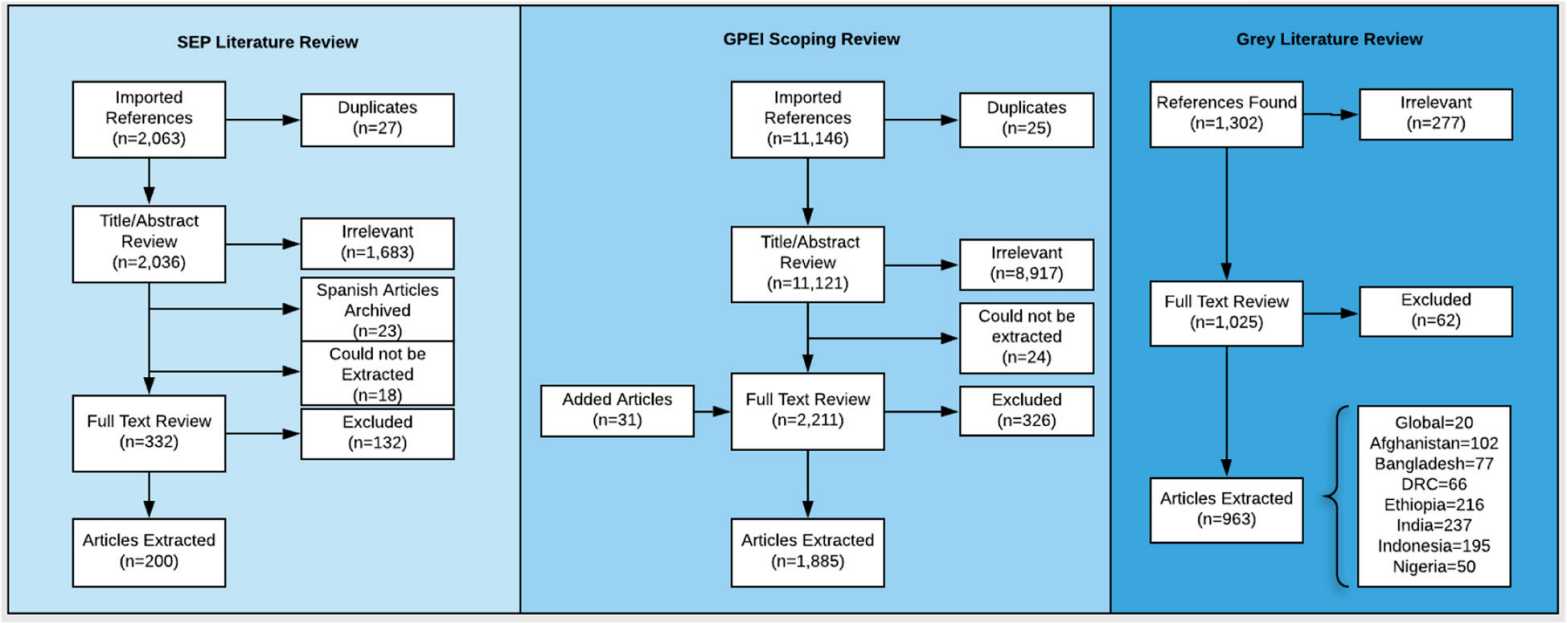
PRIMSA diagrams for three prongs of the scoping review*. *We extracted a total of 3049 articles across all three prongs of the review. We excluded articles not primary focused on polio, those focused on “upstream” such as disease physiology and vaccine development. The main reasons for not being able to extract articles are: lack of access to full text materials for some records and language limitations with included non-English articles, e.g. *Spanish and Portuguese materials*

Figure 2 outlines PRISMA diagrams for all prongs of this scoping review, including the number of articles that finally underwent full-text data extraction. Consortium teams identified different quantities and kinds of grey literature; this difference in kind of material was due to the status of polio eradication in each country and contextual factors including degree of ease in accessing repositories, conflict resulting in lack of available documentation in some countries, and also the kinds of collaborations and relationships that exist between organizations that were involved in the review and other country-level GPEI implementors.

### Full text data extraction

#### Published literature

**Fig. 3:**
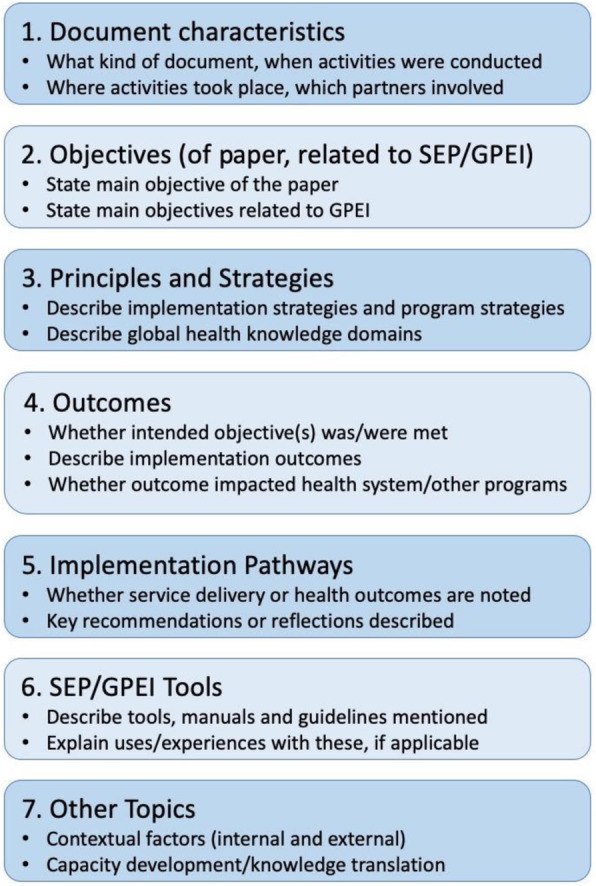
Overview of the sections of the data extraction form for all prongs of the review

For the SEP and GPEI scoping review prongs, we developed a Qualtrics^XM^-based (Seattle, WA) data extraction form based on relevant implementation science and GPEI/SEP literature and frameworks [42–45]. The tool collected information on type of article, study objectives, GPEI/SEP phases, implementation period, strategies and outcomes, global health domains, and contextual factors based on adaptation of relevant implementation science and global health knowledge frameworks (Fig. 3). Further explanation of the team-specific process for utilizing this tool is available in the Supplementary File.

For our study, implementation strategies were adopted from the Expert Recommendations for Implementing Change (ERIC) project and adapted to our specific knowledge mapping objectives [42]. We utilized the ERIC strategies because they provided a standardized set of strategies identified from implementation experiences that could enable comparison of the implementation of different programs. Furthermore, we categorized the strategies into five groupings of related strategies based on common themes such as strategies related to planning and resource allocation or communication. These categories of strategies included between three and 12 specific strategies and reviewers could choose all strategies that applied when completing the data extraction procedure. Category totals presented in the results reflect whether *any* of the specific strategies within a category were selected for a particular article.

#### Grey literature

Key documents identified for the global grey literature review were reviewed by two team members and detailed notes were taken on events, lessons learned, and illustrative quotes that would help frame the trajectory of the GPEI.

For the country grey literature review, a version of the same Qualtrics-based survey (Fig. 3) was made available to all country teams; due to connectivity challenges, a Microsoft Excel version of the tool was used by three countries (Afghanistan, Ethiopia, Nigeria) for offline data extraction. A virtual workshop to discuss the data extraction process and establish quality criteria was conducted with country teams in July 2018, and monthly meetings to review progress and discuss challenges were held through December 2018. All teams followed data extraction criteria and processes determined by centrally by the STRIPE Consortium. Within these criteria, country teams developed processes to meet data extraction timelines according to team capacity and the content of contextually relevant material.

### Data cleaning and analysis

All prongs of the review underwent the same process for data cleaning and analysis. The process is outlined in the sub-sections that follow.

#### Double reviews

We conducted double reviews for a subset of randomly selected articles, 6% of the GPEI sample and 14% of SEP sample, comparing 90 key variables out of the 342 total variables in the extraction tool. Each article was reviewed by two independent reviewers. Double reviews were not conducted in the gray literature review, except in Indonesia. For the GPEI and SEP reviews, we conducted inter-rater reliability tests to assess consistency in extraction results across multiple reviewers (further details about our methodology and results can be accessed in supplementary material for this article). Inter-rater reliability was assessed over all reviewers and across all double-reviewed articles to obtain a summary measure of agreement under the assumption of independence of extractions by the reviewers. In addition, reliability was assessed by the types of articles reviewed and by selected specific variables. Specific variables assessed included GPEI strategies, implementation strategies, global health knowledge areas, and implementation tools. Among the several inter-rater reliability statistics (including the Cohen’s Kappa and Gwet’s AC1) that were generated (Supplementary File, Table S1), [47] we chose to report the Gwet’s agreement because it is more robust to changes in prevalence ratings across categories, adjusts for the high-agreement-low-kappa paradox that is characteristic of Cohen’s and Fleiss’ kappa, and accommodates different data types found in the review [47]. All analysis was done using the *kappaetc* user-written commands in STATA (version13®, College Station, TX: StataCorp LP).

#### Qualitative coding

During the data extraction for all prongs of this scoping review, we captured free text responses on recommendations and key lessons learned in a separate field of the data extraction form. After the data extraction process was completed, these entries were coded as qualitative data in Dedoose (version 8.2.14, Manhattan Beach, CA) using a codebook that covered the WHO health system building blocks as well as inductively determined themes related to planned case studies and categories of lessons learned [48]. These themes included categories such as Advocacy and Communications as well as Program Processes, which allow us to capture underlying principles that served as barriers and facilitators in the GPEI and SEP implementation. The codebook was set-up to allow for coding of a theme as either a facilitator or barrier in each particular instance. These data were captured as counts of how many articles had included a particular theme (e.g. financing as a barrier or facilitator, or both) and also as “rich descriptions” of how these themes were described in specific contexts across the three prongs of the review.

As a foundational component of the STRIPE project, we utilized the ERIC implementation framework to categorize implementation strategies under both SEP and GPEI into five categories: planning and resource mobilization, management and problem-solving, monitoring and evaluation, engagement and capacity-building, and communication and mass media. Within each category of strategies, we explore the similarities and differences between SEP and GPEI programs and reflect on how these strategies evolved over time (see Supplementary File for additional details on the analysis conducted to inform this section of the paper).

## Results

### The quantity and scope of the three prongs of the review

In total, this scoping review included 3048 documents, for which full text data extraction was completed (Table S2 in Supplementary File). Within the GPEI published literature review (*n* = 1885), peer-reviewed journal articles and editorials combined comprised 1048 (55%) articles. Four-hundred thirty-five periodic updates published in the CDC Morbidity and Mortality Weekly Report and WHO Weekly Epidemiological Record constituted about a quarter of the articles. The GPEI grey literature (*n* = 943) included news articles, review articles, policy and policy analysis, unpublished projects, research/program protocols, and standard operating procedures. Author or respondent perspectives for the GPEI articles were primarily GPEI partners (71%), the government (52%), and the academic community (39%), and exceed 100% due to the possibility for selecting multiple perspectives during data extraction.

Within the SEP review, peer-reviewed journal articles and editorials were 122 (61%) of the the total 200 articles. Review articles, periodic updates and news articles made up the other types of documents included in the review.

Full details on the breakdown across prongs of the review by geographical distribution and author/respondent perspective can be found in the Supplementary File (Table S2). The contents of the literature in each prong of the review have distinct foci in terms of geographical focus and perspectives. For example, the SEP review included mostly global-level perspectives (143 articles, 72%)—a much higher proportion than in the GPEI (661 articles, 35%).

### Evolution of implementation strategies: linking SEP experience to the GPEI and lessons from these examples

In the following narrative, we will present examples of specific strategies commonly identified within each of five categories of implementation strategies (Table S3 in the Supplementary file). We looked for which strategies were most commonly cited within each category in order to focus on areas where significant amount of work has been conducted and where there are likely nuanced lessons to be learned. We then explored the linkage described in the conceptual framework: how experiences and learning from the SEP informed the way GPEI has thought about and utilized these implementation strategies, and how the strategies have evolved, gained complexity and specificity, and been adapted over time.

#### Planning and resource mobilization

Within the planning and resource mobilization category of strategies, the most common strategy found in the SEP literature was the use of an implementation blueprint, which includes the development of objectives as well as program activities, timelines, and performance measures (*n* = 76, 69%). While having implementation blueprints was not an explicit component of the original SEP strategy, [8] this emerged over time and was identified as essential to program success. For the GPEI, implementation blueprints and funding were part of the program strategies built into the GPEI from its inception along with the use of strategic plans and resource mobilization efforts. However, these were mentioned less frequently in the global literature (*n* = 172, 46% for each strategy) and at country level (*n* = 169, 48%). Therefore, although SEP experience led to the recognition of the value of blueprints, the GPEI did not show high fidelity in implementing these lessons.

Furthermore, implementation blueprints evolved nationally as more countries achieved polio eradication, unlike during the SEP where the implementation blueprints were more regionally-based. Ado [49] showed that in Nigeria, the implementation shifted to include a presidential task force on polio eradication, involvement of traditional leaders in the northern region of the country where implementation of polio eradication activities was a major challenge, and the set-up of emergency operations centers at the national and state levels.

Unlike under the SEP, the protracted time period under which the GPEI has been active means that different planning strategies have had to be rapidly deployed at different periods under the GPEI – and a majority of these were successfully deployed. For example, the synchronized global switch from trivalent oral polio vaccine (OPV) to bivalent OPV exemplifies a success story of strategic planning [38, 50]. According to Zipursky, [38] 105 of 126 countries using OPV only introduced IPV within a 2.5-year period, making it the fastest roll out of a new vaccine in history. This achievement was attributed to several factors, including the coordination work of the Immunization Monitoring Board, high-level engagement and advocacy across GPEI partners and also close collaboration, building on the strong foundations of the EPI at all levels, Gavi, and proactive communications with clearly defined dissemination channels. The IPV introduction can serve as a model for other vaccine introductions, especially in an accelerated context. Furthermore, Rutter et al. [51] highlighted the true independence of the GPEI Independent Monitoring Board (IMB) from the agencies and countries delivering the program which enabled it to raise difficult issues that other institutions could not. We are not aware of any literature that described the existence of such formalized monitoring boards under the SEP.

#### Management and problem-solving

Within the management and problem-solving category of strategies, the specific strategy of developing robust record systems to capture implementation and clinical outcomes was the most commonly found implementation strategy in the SEP review literature (*n* = 69, 57%), followed by adapting the program structure, e.g. evaluating and changing service delivery configurations (*n* = 58, 48%), and assessing organizational readiness (*n* = 43, 35%). This triad of strategies reflects a key learning from the SEP experience that field data, when combined with organizational flexibility and contextual responsiveness, could successfully inform programmatic adjustments and ensure feasibility. One author noted SEP’s success hinged on partners’ ability to appropriately define potential implementation barriers and change operational approaches as experience accumulated, a capacity which relied upon adequate and appropriate data systems to capture program feedback [52]..

The development of robust record systems was a key learning carried forward into the GPEI. Among all related strategies cited in the GPEI literature, developing robust record systems was also the most commonly identified implementation strategy (*n* = 246, 48%). Maintaining the focus on data as a primary management tool, actors involved in polio eradication developed new approaches to the kind of records that were kept, including community mapping and microplanning, and strategies to mark houses for program monitoring. These strategies were particularly important for improving coverage among hard-to-reach communities, which often faced challenges related to enumeration and accessibility. Major lessons around assessing organizational and health systems readiness to identify implementation barriers prior to rolling out core strategies were infrequently applied in GPEI (*n* = 213, 48%) compared to the SEP. The operational flexibility by which record systems were set up and used under the SEP were not initially carried along into GPEI, however, given the GPEI’s centralized nature.

#### Monitoring and evaluation

Among the monitoring and evaluation strategies, having specific mechanisms for feedback to relevant stakeholders in order to modify and evaluate programming was the most common strategy identified within the SEP (*n* = 96, 97%). The importance of periodically re-evaluating the performance of strategies deployed and creating a feedback loop to adjust the strategies accordingly came to the fore as the Smallpox Intensified Eradication Program commenced. Supervisory and containment teams, headed by epidemiologists, were mobilized across administrative levels to facilitate quick detection and rectification of deficiencies as well as redeployment of scarce resources. Collective review and feedback from field staff resulted in several innovations, such as the development of recognition cards with a picture of a child showing a typical smallpox rash to be used for eliciting information of new cases or aid with weekly searches at markets, fairs, schools, etc. [12]

The GPEI also commonly noted the importance of feedback mechanisms (*n* = 348, 90%), and instituted these mechanisms across multiple levels. Emphasis on involving stakeholders representing different areas of expertise, to allow for independent monitoring and oversight as well as maintaining transparency and accountability was placed early on in the program. For example, the introduction of checklists for front-line workers, along with supportive supervision to enable checks on data accuracy, helped reach missing key child populations once data were analyzed and applied to the program. The introduction of lot quality assurance sampling to monitor supplementary immunization activities (SIAs) in endemic countries helped identify gaps in SIA quality and high-risk areas which led to marked improvement in SIA quality [49, 53]. Similarly, at country level (*n* = 290, 80% in the grey literature) in Nigeria, surveillance activities relied on a data sharing and feedback process including the laboratory network, the federal government, the WHO, and the national polio emergency operations center in order to monitor laboratory quality as well as rapid notification of poliovirus detection [54].

#### Engagement and capacity building

Under engagement and capacity building-related strategies, involving stakeholders—including existing governance mechanisms, communities and other key partners—in the implementation effort was the most common strategy identified in the SEP literature (*n* = 95, 73%). Within GPEI (*n* = 289, 53%), this strategy took on additional levels of complexity and variability across contexts as the program worked to adapt implementation approaches and respond to other challenges [55]. These plans included holding stakeholder engagement events in each country within the GPEI; these stakeholders then remained involved through testing of implementation dashboards, monitoring and evaluation plans, and roll-out of the switch itself [56]. The country level grey literature also frequently mentioned stakeholder engagement (*n* = 298, 58%).

#### Communications and mass media

Finally, under the communication and mass media category, increasing awareness among target populations was the most common specific strategy in the SEP (*n* = 48, 92%). Within the GPEI, this strategy remained critical, particularly in terms of reaching marginalized populations (*n* = 261, 90%). The Rotary International’s PolioPlus Program demonstrated the importance of civil society partnerships in global health and international advocacy. Rotary’s polio eradication efforts raised funds with coordinated campaigns, and raised awareness with innovative communication methods and celebrity engagement at global and national levels [57]. The reviewed SEP literature did not emphasize these innovative communication methods as compared to the GPEI, and this may reflect the changing global social context and the increased significance of communication and media in influencing health and wellbeing at various societal levels over time. Further, communicating the need for multiple rounds of vaccinations and the virus strain the vaccination is targeting is needed to reduce vaccination fatigue and hesitancy [58, 59]. The country-level grey literature also frequently mentioned the importance of raising awareness including awareness of polio activities, benefits of immunization, or sharing information to address concerns that were resulting in vaccine hesitancy (*n* = 330, 76%).

## Discussion

This review aimed to capture the experience of implementation of the SEP and GPEI initiatives, focusing on the relative importance of implementation strategies between the programs and how these strategies have evolved as the breadth and depth of experience builds. We have begun to draw out lessons that other global health initiatives can follow, and we will further discuss those in this section. Based on our exploration, we found several strategies that were not included explicitly in the SEP program plans but were found to be important during the program’s lifecycle and became prominent in GPEI. We also found a number of “new” strategies that were not explicitly part of the SEP strategy, but were emphasized within the GPEI.

Within the Planning and Resource Mobilization category of strategies, we found that implementation blueprint was a frequently mentioned aspect of the SEP that also featured in the GPEI. Within the GPEI, the level of sophistication and specificity of these blueprints shifted. For example in India, while a national blueprint (based on global guidelines and South East Asia Regional Office (SEARO) negotiations) had been established for polio eradication—which included mass immunization as well as Pulse Polio initiatives—inadequate resources were provided to implement the blueprint including the understood need to strengthen the overall routine immunization system in India [60]. This reality contributed to both frustration among implementers in country as well the longer timeframe needed to achieve certification for the country [60]. Further, while funding was a challenge to both programs at times [8, 27, 61], emphasis on fundraising as a strategy to achieve implementation outcomes was found more commonly in the GPEI literature. Finally, in terms of strategic planning, other global health programs might benefit from establishing similar independent monitoring mechanisms, overcoming the IMB’s limitations and building on its strengths.

Management and Problem-solving strategies shared by both the SEP and GPEI included the development of robust record-keeping systems, which was identified through the experience of the SEP implementation and carried forward into the GPEI. Within the GPEI, in response to the recognized need to target specific sub-populations, mapping and household enumeration strategies were integrated into program implementation. Specifically, within the grey literature from country level, the need to ensure organizational capacity and readiness also came up repeatedly for the GPEI. For example, the CORE Group utilized localized planning and social mobilization activities in Uttar Pradesh, India in order to improve supplemental immunization and worked to capture record keeping systems that could help them track the impact of these interventions [62, 63]. Other global health programs can build off of this implementation experience to proactively establish these collaborative and accountable management strategies into their programs.

Monitoring and Evaluation strategies across the SEP and GPEI both included developing mechanisms for providing feedback. Its importance was identified during the final stages of the SEP, as campaigns intensified to reach the final strongholds of smallpox and countries shifted strategies, moving from mass vaccination to surveillance and containment. The strategy was subsequently embedded in the implementation of the GPEI, wherein processes were monitored at every phase of the program, including the introduction and withdrawal of vaccines, accuracy of data collected, and synchronized immunization days. Adjusting to changing political, economic, social, and technological contexts, mechanisms for the collection and sharing of experiences of implementing the GPEI were highly varied and nuanced. For example, monitoring took place before, during, and after campaigns via daily meetings to identify and appropriately escalate process issues, review and validate tally sheets to spot discrepancies, as well as assess finger and house markings to appropriately plan effective follow up measures. While there has been substantial learning in this area, there have also been challenges in terms of the timeliness of feedback, how feedback is used, and how decentralized feedback and decision-making processes are. Other global health programs can similarly build mechanisms for completing robust periodic assessments of current strategies and creating feedback loops to all stakeholders allowing for necessary iterations and corrective measures in real-time.

Strategies for Engagement and Capacity Strengthening included stakeholder engagement for both the SEP and GPEI, though a lower proportion in GPEI (53%) than in the SEP literature (73%). GPEI innovated in this area, including strategically engaging youth, establishing national level committees for traditional and religious leaders, and setting up of presidential-level committees and strategic partnerships for national and international oversight of GPEI implemenation. For example, in countries in the South-East Asia region, stakeholders’ engagement plans were made for each of the 11 countries that would be making the switch from trivalent to bivalent OPV [56]. Musa et al. described the innovative strategic engagement of youth groups to reduce team harassment during vaccination campaigns and improve vaccination coverage in non-compliant communities in Kaduna State, Nigeria [64] while Gammino et al’s evaluation highlighted the importance of deconstructing the mechanisms of rejection of polio immunization required the GPEI to work with traditional and local government leadership to systematically identify non-compliant households, block rejection areas, identify the most salient concerns and address them [65]. Other global health programs can also integrate stakeholder engagement across all aspects of program implementation from planning through to evaluation and consider which level(s) are most appropriate for specific programs and contexts.

Finally, for Communications and Mass Media, the SEP increased awareness about the importance of this strategy. Within the GPEI, understanding of the role of communications evolved to emphasize reaching marginalized populations, including at national and sub-national levels. At country level, for example in Nepal, the importance of raising awareness among parents about when National Immunization Days (NID) would be taking place and whether their children needed to participate was high priority [66]. While most parents in Nepal (over 90%) were aware of the NID, awareness about the reason for the program was quite low (24.4%) and additional strategies to raise awareness of parents in poor, rural settings, with limited education, were identified to be necessary [66]. Global health programs going forward can integrate this experience by planning for the most effective role for communications, particular as the use of mobile devices and connectivity continues to expand around the world.

### Strengths and limitations of this scoping review

The quantity and content of material that we included was larger and more diverse than we originally anticipated. As we have continued to utilize the findings from this review to inform our work as well as serve as the basis for more focused papers, we have recognized that the breadth of the review has strengths and weaknesses. The strengths are the comprehensiveness and the rich data on implementation aspects (strategies, tools, outcomes). However, what we have found as we work on papers and curricula across much more specific topics within the SEP and GPEI experience, is that the initial broad swath of variable that we captured in the review provides an overview of how frequently various topics and approaches were mentioned and some summary information on each, but does not always provide an ideal level of nuance and specificity for gaining in-depth understanding of a focused topic of interest (e.g. community engagement approaches at district and local levels or for understanding how negotiation and dialogue have been utilized within the GPEI) without going back to that sub-set of literature and extracting further details related to the focused topic of interest.

This scoping review faced several challenges and limitations. Despite the large quantity of materials that we captured, given the breadth and duration of the GPEI and SEP initiatives and variations in archival processes, we were not able to capture every relevant document. In order to ensure that we captured as much relevant material in an organized and easy-to-use format as possible, we created an online database that includes full text versions of all documents in this review as well as additional collections of materials identified that either did not fit within the scope of planned review, could not be reviewed due to time ore resource limitations (additional grey literature, non-English literature), or were identified after we completed data extraction in order to be able to review, reference, and draw upon specific collections of additional material for the project moving forward. There are additional databases that were not included in the GPEI review that may have added some additional learnings, and there is additional grey literature at global level, in other countries that were not included in this project, as well as likely within countries that were included but which were not accessible to our teams. Our choice to exclude “upstream” articles means that these data sets do not capture all of the relevant vaccine science that informed SEP and GPEI program strategies. In addition to these limitations, we also faced several challenges and limitations as we were conducting the review. These included reviewer fatigue and time constraints, particularly given competing priorities within and outside the STRIPE project.

Finally, the results of the interrater reliability analysis indicated how the reviewers evolved in their agreement over the review period. While initial agreement was moderate, it became almost perfect among raters of several articles over time. The GPEI and SEP reviews have somewhat different agreement, with the SEP review having slightly higher rates of agreement overall, including up to perfect scores. This is likely due to several factors, one of which is that this review was conducted after the GPEI review when many of the reviewers were quite seasoned. In addition, there were fewer reviewers and the content of the SEP literature may also have been slightly less complex (for example, the number of SEP strategies that were extracted for was much smaller than for the GPEI).

## Conclusion

Within this review, we have explored the kinds of learning that take place within global health programs over time, using the SEP and GPEI as an example. We have seen that global health programs do learn from each other and evolve in response to changes in context and over time. Key lessons from the SEP and GPEI implementation include: 1) In addition to overarching or generic strategies that a program plans to or learns it needs to use, there is a need to embrace and incorporate nuance and adaptation to time, place, and context, and 2) related strategies (e.g. different strategies for resource mobilization) were not always identified in the literature over time, or always used consistently leading to potential for missed opportunities for learning and impact. Strategies and their implementation in a global health program should be well codified or described, including information on the contextual, time and stakeholders’ issues that these strategies respond to.

From the experience of undertaking this review, we also became increasingly convinced that global health programs can continue to learn from each other’s implementation experiences more effectively when this experience is captured and made available in user-friendly formats. The quantity, diversity, and spread of the materials we identified, screened, and included in the review would be really challenging for other stakeholders such as practitioners and many leaders to systematically process in order to glean best practices and recommendations from. In addition,

when undertaking broad scoping reviews such as this one, ensuring that the resulting data set includes sufficiently focused and developed data to inform nested more specific analyses is key. Otherwise, when undertaking a more focused analysis, there is limited value-add to starting from the large scoping review dataset rather than just doing a new more focused search.

Going forward, a core component of the STRIPE project is to develop resources and a curriculum for teaching implementation science and opportunities for other global health programs to learn from the SEP and GPEI implementation experience. Through this core curriculum, which will be adapted and implemented across diverse country contexts as well as presented as a massive open online course (MOOC), STRIPE will utilize the experiences from GPEI and SEP to teach implementation science concepts and their application in global health programs. Therefore, these data will be able to directly information program implementation as well as contribute to the learning of the next generation of implementation scientists.

## Supplementary information

**Additional file 1.** Details About the Methods for Selecting Documents for this Review.

## Data Availability

These data will be made available as open access datasets by the end of the project period.
