# Supplementary material for: A multi-pronged scoping review approach to understanding the evolving implementation of the Smallpox and Polio eradication programs: what can other Global Health initiatives learn?
Source: BMC Public Health. 2020 Dec 18;20(Suppl 4):1698. doi: 10.1186/s12889-020-09439-1 (PMC7747000; doi:10.1186/s12889-020-09439-1)
Supplement: Supplementary file 1 — Additional file 1. Details About the Methods for Selecting Documents for this Review. [file 12889_2020_9439_MOESM1_ESM.docx]

**Supplementary Material**

**Additional Details About the Methods for Selecting Documents for this Review**

**Search Strategy**

For all prongs of the review, we initially planned to build a comprehensive set of search terms that captured all aspects of interest including implementation strategies, tools, and lessons learned. However, after multiple iterations, we were not able to create a set of search terms that captured a set of index articles fully without the terms being so broad and inclusive that they no longer provided specificity. Therefore, our final approach for the published literature was to capture all search results for smallpox and polio. The SEP search terms were: “smallpox[MeSH] OR "small pox"[tiab] OR "smallpox"[tiab] OR Variola[tiab] OR Variolas[tiab] OR Alastrim[tiab]” and limited to data from January 1, 1950 till May 7, 2018. The polio search terms were: "Poliomyelitis"[Mesh] OR "Poliomyelitis, Bulbar"[Mesh] OR Poliovirus[tiab] OR Polio[tiab] OR "infantile Paralysis"[tiab] OR "acute flaccid paralysis"[tiab] NOT ("animals"[MeSH Terms] NOT ("humans"[MeSH Terms] AND "animals"[MeSH Terms]))” and limited to publications from January 1, 1988. The cut-off for the GPEI published literature was April 25, 2018 and for the SEP was May 7, 2018. We applied further inclusion/exclusion criteria during the title/abstract screening phase.

For the grey literature search, we searched GPEI and core partner websites as well as sourced materials from key informants. For grey literature review at country level, teams in the seven STRIPE partner countries were also identified in May 2018. A virtual workshop reviewing grey literature review processes and the objectives of the review was conducted on May 30, 2018. Each country team devised a search strategy tailored to their particular context, but which consistently included archives and repositories of GPEI core partners in country, other large archives or collections, online searches using Google and specific online repositories, and documents identified while interviewing key informants for another portion of this project. Each team conducted a search from June 2018 to February 2019 utilizing multiple strategies, including meeting with key GPEI partners in country to request documents, accessing governmental archives and other relevant collections, and conducting online searches for country-specific GPEI documents. Three countries (Ethiopia, Bangladesh, and Afghanistan) added additional documents to their collections in May and June 2019 based on discussions with project partners to fill identified gaps.

**Inclusion/Exclusion Criteria and Title/Abstract Screening**

*Published Literature:* Covidence (Melbourne, Australia) systematic review software was used to screen title and abstracts for both the GPEI and SEP scoping review. Two reviewers independently screened each article. Conflicts in decisions to include or exclude were resolved by discussing the reasoning for the decision, or by a third reviewer with substantial background in the topic area providing final determination for areas of discordance. We developed a tagging system, with tags such as “laboratory studies” and “vaccine development,” for all titles and abstracts in order to gain an understanding of the scope of the SEP and GPEI search results as a whole, including materials that we excluded from this scoping review.

Following the title/abstract review, we obtained full text materials for all articles for which data extraction was to be completed. For the GPEI scoping review, full text for included references were searched for via EndNote, connected with the William Henry Welch Library. Full text articles not found in EndNote were searched for manually via PubMed. Full text articles not found via PubMed were requested via WelDoc inter-library loans at the Welch Library, Johns Hopkins University. Remaining full text articles not retrieved via WelDoc were requested a second time to account for any processing errors. A total of 23 full text articles could not be found. Reasons articles were not found included retrieval errors by partner libraries, delayed responses to materials requested by partner libraries, and inability to find citations as retrieved from original searches.

For the SEP scoping review, full text for included references were also searched for via EndNote, and those not automatically retrieved were searched for manually via PubMed, SCOPUS, and EMBASE. Full text articles not found via manual searches were requested via WelDoc loans at Welch Library, and a second request to WelDoc was submitted for articles that were not found on the first round. A Welch informationist was consulted for further tips on article searching. Kopernio was downloaded and used to search for full text of remaining articles based on informationist suggestion. A total of 10 full text articles could not be found. Reasons articles were not found included very old articles (due to inclusion criteria) that could not be found or were not retrieved in time for review.

*Grey Literature:* The country-level grey literature search results were reviewed to determine any overlap with the global SEP and GPEI scoping reviews; any overlapping articles were reviewed by the JHU team as part of the scoping reviews and were excluded from reviews at the country level. Country teams were requested to prioritize materials only available at the country level. They worked to access full text materials directly from partner organizations and government entities; no significant challenges in obtaining full-text documents occurred where a relevant citation was recommended to teams or identified through searches.

**Full Text Data Extraction**

In preparation for data extraction, all team members received background documents on the GPEI, the overall study aims, and some seminal literature on implementation research (17, 19, 20, 22-24) that informed the data extraction tool development. A pilot test was undertaken (three articles for SEP and nine articles for GPEI) and the results were discussed as a team, particularly areas of divergence. Following the pilot test, the team met once a week throughout the duration of the data extraction period to review challenges, grey areas, and other questions that arose. Discussions during these weekly meetings helped solidify a shared understanding of project aims and the purpose of each question in the data extraction form; this ensured a common approach, though we recognize that perfect alignment of responses for some of the more complex questions with many answer options were not feasible.

**Data Cleaning:**

For each stream, data collected across teams was compiled into a single dataset in R (version 3.3.2). This raw data generally contained responses that corresponded with more than one answer choice due to the nature of the data extraction tool which allowed data collectors to enter more than one response to a single question. Dummy variables were created for each potential response in R and assigned accordingly. Qualitatively derived codes were also merged to their respective datasets in R. Finalized datasets were then converted into Stata .dta files for further analysis.

**Summary of Interrater Reliability Methods and Results**

**Methods**

Sample size estimation for interrater reliability: Using the *sskdlg* user-written commands (ado file) in Stata 13®, we estimated the sample size required for the interrater agreement using two independent raters. We used a precision-oriented perspective which allows us to generate confidence intervals around our kappa statistics of 100(1-alpha)% for the standard errors of the estimates. All analysis was done using the *kappaetc* user-written commands (ado file) in Stata 13®.

**Results**

Sample size estimation: The sample size estimated for an expected kappa of 80% or above signifies almost perfect agreement. Using a conservative probability of positive rating by each reviewer at 50% with 10% precision around the estimates and 95% confidence interval yielded a total sample size of 135 articles for each of the GPEI and SEP reviews.

Sample size was varied along the following parameters:

**Table S1: Summary of the Results of he Sample Size Estimation**

| Expected Kappa | Probab. Positive by rater 1 | Probab.positive by rater 2 | Absolute precision | Estimated sample size |
| --- | --- | --- | --- | --- |
| 0.8 | 0.5 | 0.5 | 0.05 | 553 |
| **0.8** | **0.5** | **0.5** | **0.10** | **135** |
| 0.9 | 0.5 | 0.5 | 0.05 | 292 |
| 0.9 | 0.5 | 0.5 | 0.10 | 73 |

For the GPEI review, 138 articles were selected and 132 had valid results. For the analysis, we excluded six articles for: 2 articles for invalid responses (articles 410 and 1240) and other four had no reviewer ID (#417, 536, 1207, 1528). For the SEP review, a total of 27 articles (14% of all articles) were reviewed.

**Results of Agreement Statistics for GPEI and SEP Reviews**

Among the several inter-rater reliability statistics that were generated, we chose to report the Gwet’s agreement because it is more robust to changes in prevalence ratings across categories, adjusts for the high-agreement-low-kappa paradox that is characteristic of Cohen’s and Fleiss’ kappa, and it  accommodates different data types found in the review. Since different articles were reviewed by different sets of reviewers and not all reviewers reviewed all articles for the double reviews, estimating an overall percent agreement would be invalid. Instead, we report a range of percent agreement statistics for the different sets of articles and sets of reviewers that rated the articles. For the GPEI review, the double review comprised of 132 articles by 9 individual raters across 90 variables in the abstraction tool. At least two raters reviewed each article.  Across selected articles, percent agreement of raters ranged from 0.48 (95% CI: 0.38-0.58) to 0.91 (95% CI: 0.85-0.97).  The chance-corrected Gwet’s agreement coefficient among raters ranged from 0.41 (95% CI: 0.29-0.53) to 0.90 (95% CI: 0.84-0.97). The results indicated how the reviewers evolved in their agreement over the period of the review where initial agreement was moderate and became almost perfect among raters of several articles over time. For the SEP review, the double review comprised of 27 articles by 6 individual raters across 73 variables in the abstraction tool. Two raters reviewed each article.  Across selected articles, percent agreement of raters ranged from substantial agreement of 0.60 (95% CI: 0.48-0.72) to 1.0, indicating perfect agreement.  The chance-corrected Gwet’s AC among raters ranged from 0.57 (95% CI: 0.44-0.70) to perfect agreement, indicating moderate to perfect agreement across raters.

**Additional Details Regarding the Quantity and Scope of the Scoping Review Prongs**

The Table 2 below details the quantity and scope of the three prongs of this scoping review.

**Table S2: Scope and Characteristics of the Data**

| Characteristic | SEP Review  N=200 (%)^#^ | GPEI Review  N=1,885 (%)^#^ | Grey Literature  N=963 (%)^#^ |
| --- | --- | --- | --- |
| **Geographical Distribution** | | | |
| Global | 143 (71.5%) | 661 (35%) | 20 |
| WHO Africa Region | 46 (23%) | 744 (39.5%) | - |
| WHO Eastern Mediterranean Region | 26 (13%) | 640 (34%) | - |
| WHO European Region | 7 (4%) | 196 (10%) | - |
| WHO Southeast Asia Region | 67 (34%) | 655 (35%) | - |
| WHO Western Pacific Region | 3 (1.5%) | 213 (11%) | - |
| WHO Region of the Americas/PAHO | 27 (14%) | 167 (9%) | - |
| Afghanistan | 7 (3.5%) | 340 (18%) | 102 (11%) |
| Bangladesh | 26 (13%) | 84 (4.5%) | 77 (8%) |
| DRC | 143 (8%) | 143 (8%) | 66 (7%) |
| Ethiopia | (21 (10.5%) | 120 (6%) | 216 (22%) |
| India | 53 (26.5%) | 597 (32%) | 237(25%) |
| Indonesia | 10 (5%) | 62 (3%) | 195 (20%) |
| Nigeria | 11 (5.5%) | 513 (27%) | 50 (5%) |
| **Author / Respondent Perspective** | | | |
| GPEI partner organizations* | - | 922 (49%) | 328 (35%) |
| WHO/PAHO | 63 (32%) | - | - |
| Academic / research institution | 111 (56%) | 804 (43%) | 62 (7%) |
| Government | 34 (17%) | 249 (13%) | 292 (31%) |
| CSOs | 0 | 65 (3%) | 66 (7%) |
| NGOs | - | - | - |
| Consulting firm | 2 (1%) | 34 (2%) | 15 (2%) |
| Implementing organization | 2 (1%) | 40 (2%) | 113 (12%) |
| Funding organization | 2 (1%) | 30 (2%) | 118 (13%) |
| Other | 8 (4%) | 182 (10%) | 24 (3%) |

*^#^Columns do not add up to 100% because more than one option was allowed for responses.*

* World Health Organization (WHO), the US Centers for Disease Control and Prevention (CDC), Rotary International, UNICEF, and the Bill and Melinda Gates Foundation

**Frequency Analysis Across Categories of Implementation Strategies**

Our data extraction tool included questions on multiple implementation science components. Our Phase 1 analysis, however, focused on mapping implementation strategies across the five categories developed at the data extraction phase (Table 3). Totals for each category of strategy mean the number of times a document mentioned *any* of the specific strategies within a category. We also began to explore how the use of strategies has evolved from the SEP to the GPEI and examples of how these strategies showed up at country level.

**Table S3: Results of the Scoping Review Findings Mapped to Implementation Strategies**

| **IS Category** | **SEP (N=200)** | **GPEI (N=1,885)** | **Grey Literature (N=943)** |
| --- | --- | --- | --- |
| **Planning and Resource Mobilization** | **(N=110)** | **(N=376)** | **(N=352)** |
| Implementation blueprint | 76 (69%) | 172 (46%) | 169 (48%) |
| Funding | 44 (40%) | 172 (46%) | 72 (20%) |
| Service site | 35(32%) | 111 (30%) | 123 (35%) |
| Scale-up | 23 (21%) | 60 (16%) | 133 (38%) |
| **Management and Problem Solving** | **(N=122)** | **(N=516)** | **(N=444)** |
| Organizational readiness | 43 (35%) | 111 (22%) | 213 (48%) |
| Adapt structure | 58 (48%) | 84 (16%) | 132 (30%) |
| Record systems | 69 (57%) | 246 (48%) | 148 (33%) |
| Model and simulate | 23 (19%) | 87 (17%) | 131 (30%) |
| Dissemination organization | 33 (27%) | 55 (11%) | 66 (15%) |
| Centralized assistance | 28 (23%) | 74 (14%) | 99 (22%) |
| Incentives & disincentives | 30 (25%) | 105 (20%) | 21 (5%) |
| **Monitoring and Evaluation** | **(N=99)** | **(N=388)** | **(N=364)** |
| Mechanisms for feedback | 96 (97%) | 348 (90%) | 290 (80%) |
| cyclical small tests of change | 3 (3%) | 18 (5%) | 30 (8%) |
| Credentialing & liability standards | 21 (21%) | 43 (11%) | 96 (26%) |
| Visit other sites | 2 (2%) | 25 (6 %) | 36 (10%) |
| **Engagement and Capacity Building** | **(N=130)** | **(N=550)** | **(N=512)** |
| Multidisciplinary Partnerships | 75 (58%) | 187 (34%) | 212 (41%) |
| Leverage network collaborations | 68 (52%) | 192 (35%) | 181 (35%) |
| Workshops | 26 (20%) | 143 (26%) | 98 (19%) |
| Involve stakeholders | 95 (73%) | 289 (53%) | 298 (58%) |
| Local knowledge | 29 (22%) | 162 (29%) | 141 (28%) |
| Dynamic and varied training | 4 (3%) | 33 (6%) | 43 (8%) |
| Recruit and train leaders | 14 (11%) | 76 (14%) | 62 (12%) |
| Train trainers | 0 (0%) | 22 (4%) | 24 (5%) |
| Supervision | 19 (15%) | 98 (18%) | 136 (27%) |
| Expert management & use data | 18 (14%) | 105 (19%) | 61 (12%) |
| Shift roles of providers | 15 (12%) | 27 (5%) | 27 (5%) |
| Learn from experts | 10 (8%) | 66 (12%) | 26 (5%) |
| **Communication and Mass Media** | **(N=52)** | **(N=289)** | **(N=436)** |
| Mass media | 17 (33%) | 109 (38%) | 180 (41%) |
| Champions | 9 (17%) | 77 (27%) | 151 (35%) |
| Increase awareness | 48 (92%) | 261 (90%) | 330 (76%) |

**Analysis Plan:**

Our analysis plan for these data from the scoping review prongs includes several phases. We aimed to provide descriptive statistics, explore associations between variables, and compare findings across prongs of the review. Following the data cleaning phase, we conducted exploratory data analysis including univariate analyses of all key variables (perspectives, target audiences, countries, GPEI/SEP and implementation strategies, outcomes, tools, lessons learned) to describe the scope and content of the findings across key aspects of the STRIPE project (Phase 1).

In the upcoming phase of analysis, we plan to explore specific aspects of the findings, such as implementation strategies and tools, manuals, and guidelines, in more depth. We will also explore key implementation pathways from implementation strategies leading to implementation outcomes and finally to service outcomes and impact.(20) During this phase, the scoping review will also contribute to a series of case studies on topics of interest such as “hard-to-reach” populations, conflict and insecurity, and the switch from OPV to IPV, which will be utilized in an upcoming course on implementation research in global health. Finally, we plan to undertake comparative analysis looking across the prongs to compare and contrast findings across different prongs.
